# A systematic review of the diagnostic aspects and use of *Trypanosoma rangeli* as an immunogen for *Trypanosoma cruzi* infection

**DOI:** 10.1590/0037-8682-0608-2019

**Published:** 2020-09-11

**Authors:** Taciana de Souza Bayão, Marli do Carmo Cupertino, Nicholas Alfred Joseph Mayers, Rodrigo Siqueira-Batista

**Affiliations:** 1 Universidade Federal de Viçosa, Departamento de Medicina e Enfermagem, Laboratório de Métodos Epidemiológicos e Computacionais em Saúde, Viçosa, MG, Brasil.; 2 Faculdade Dinâmica do Vale do Piranga, Escola de Medicina, Ponte Nova, MG, Brasil.; 3 Universidade Federal de Viçosa, Departamento de Medicina Veterinária, Viçosa, MG, Brasil.

**Keywords:** Trypanosoma rangeli, Trypanosoma cruzi, Vaccines, Diagnosis

## Abstract

**INTRODUCTION:**

*Trypanosoma rangeli* is a protozoan that infects several domestic and wild mammals and shows significant distribution in Latin American countries. *T. rangeli* infection is similar to Chagas disease, both in diagnostic and prophylactic terms. Thus, the objective of this work was to review the diagnostic aspects and use of *T. rangeli* as an immunogen for *Trypanosoma cruzi* infection.

**METHODS:**

For this elaboration, Preferred Reporting Items for Systematic Reviews and Meta-Analyses (PRISMA) guidelines were adopted with descriptors derived from the Medical Subject Headings (MeSH) platform in the PubMed/MEDLINE and SciELO databases. The inclusion criteria were defined as original articles on "*Trypanosoma rangeli*" and diagnostic aspects of *T. rangeli* infection in humans and/or research on the possible vaccines developed using *T. rangeli* strains for *T. cruzi* infection.

**RESULTS:**

After applying the inclusion and exclusion criteria, 18 articles were procured, of which 4 addressed research on the possible vaccines developed using *T. rangeli* for *T. cruzi* infection in vertebrates and the remaining 14 predominantly dealt with the diagnostic aspects of *T. rangeli* infection in humans.

**CONCLUSIONS:**

In this study, we formulated a compilation of the essential literature on this subject, emphasizing the need for more accurate and accessible techniques for the differential diagnosis of infections caused by both protozoa, and underscored several prospects in the search for a vaccine for Chagas disease.

## INTRODUCTION

*Trypanosoma rangeli* ^1^, a protozoan of the family Trypanosomatidae, is a microorganism of wide geographical distribution throughout the Central and South American regions^2^ ^,^ ^3^. Primates and rodents are some of the vertebrate reservoirs for *T. rangeli* ^4^ ^,^ ^5^. Hematophagous insects, such as the triatomine vector *Rhodnius prolixus*, can propagate the protozoan while feeding on these vertebrate hosts. This can provoke intense immune response, producing high levels of antibodies in the infected species^6^. *T. rangeli* infection stands out as a differential diagnosis for *Trypanosoma cruzi* (etiologic agent of Chagas disease) infection due to overlapping geographical distribution of the causative microorganisms, allowing the existence of simple and/or associate infections in both invertebrate and vertebrate hosts^2^ ^,^ ^7^.

The protozoan *T. rangeli*, when presented in blood cultures of vertebrate hosts, possesses an undulating membrane of length 34 μm from one end to its free flagellum (*T. cruzi* membrane is more pleated)^3^. It has a small ovoid nucleus and a kinetoplast situated at a short distance from its posterior extremity. Nevertheless, considering only the morphological parameters, the characteristic elevated pleomorphism of *T. rangeli* makes it difficult to distinguish it from *T. cruzi* ^3^ ^,^ ^8^ ^,^ ^9^. In recent decades, studies on the molecular aspects, including genome and transcriptome, of both species have contributed to the increased awareness of their tripanolytic factors within their vectors and the pathogenic capacity of *T. cruzi* in mammals^4^ ^,^ ^10^.

The period of spontaneous resolution of *T. rangeli* infection in human hosts has been estimated to be approximately 18 months^11^. However, even though this infection in mammals can result in low parasitemia and absence of clinical complications, investigation of the induced immune response in the hosts and evaluation of the development, lifespan, degree, and protection of the possible *T. cruzi* antibodies are of great significance^2^ ^,^ ^6^. The strategies for the development of vaccines against *T. cruzi* have prophylactic potential and broad medical relevancy because Chagas disease is a parasitic infection with a high mortality rate in humans, notably in the Americas, showing an estimation of 10,000 deaths annually. Moreover, its main clinical manifestation, chronic Chagas cardiomyopathy (CCC), can be particularly lethal in affected individuals. Recent studies have indicated that this disease has affected approximately 7 million people^2^ ^,^ ^12^ ^,^ ^13^.

Thus, due to the geographical overlap and morphological and immunological similarity of these two protists, it can be inferred that there is a need for techniques that allow the correct diagnosis of suspected Chagas disease cases. Therefore, the objectives of this article were to (i) address the diagnostic differentiation of *T. cruzi* and *T. rangeli* infections and (ii) investigate the capability of *T. rangeli* in developing a vaccine for Chagas disease.

## METHODS

**FIGURE 1:**
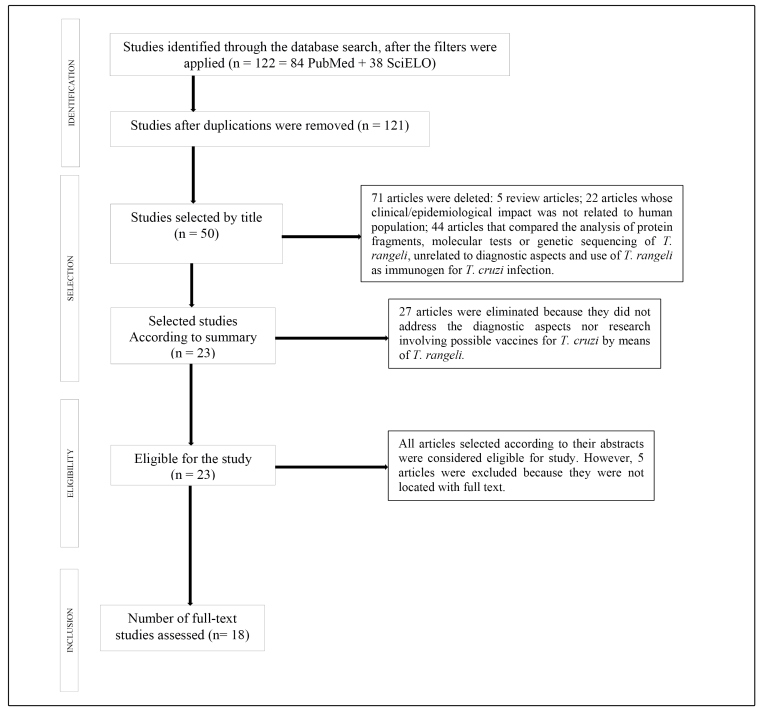
Flow diagram of the review survey results, based on items from preferred reports for Systematic Reviews and Meta-Analyzes: The PRISMA Statement^14^. The methodological tool applied in this research is detailed above. After using the exclusion and inclusion criteria, 18 articles were selected. Such texts were grouped into two thematic lines: 14 articles with an approach centered on epidemiology and diagnostic methods of the two protozoa and 4 articles including research on the possible vaccines developed using *Trypanosoma rangeli* for *Trypanosoma cruzi* infection.

We used the Preferred Reporting Items for Systematic Reviews and Meta-Analyses (PRISMA) guidelines for the preparation of this review, as shown in Figure 1 ^14^. We searched for original articles, published until October 2018, on the digital platforms, PubMed/MEDLINE and SciELO, and selected and evaluated them for a systematic review. The search filter was developed according to the platform's thesaurus, Medical Subject Headings (MeSH) terms, with the descriptor "*Trypanosoma rangeli*" identified in the "Title”, and a "human infection" filter was explicitly delimited on the PubMed/MEDLINE platforms. On the SciELO platform, the same descriptor and filters that were specific to the platform, "Health Sciences”, "Tropical medicine”, and "Infectious Diseases”, were used in Thematic Areas. We emphasize that although the search strategy included "human infection”, the articles with non-human animal models were also considered for further evaluation because some of these texts showed clear epidemiological, clinical, diagnostic, or prophylactic aspects of *T. rangeli* infection in humans.

There was no elaboration of the chronological or language restrictions in either of the platforms used. Duplicate reviews and studies were removed after conferring the authors, titles, years, and publication journals. When queries arose, the particular publications were downloaded and evaluated. The articles were initially selected through the analysis of several ‘Titles’ and ‘Abstracts’, according to the data presented in Figure 1. 

The inclusion criteria included (i) original articles, case reports and series, and clinical trials and (ii) experimental studies using *in vitro*, human, and murine models and showing implications for human studies, such as vaccine-related or clinical/epidemiological/prophylactic studies. After the assessment of the ‘Titles’ of the non-duplicated screened studies, the exclusion criteria were used to classify the included studies into (i) review articles; (ii) articles in which the clinical/epidemiological effects were not related to human populations; and (iii) articles that sought protein fragment analysis, molecular assays, or genetic sequencing of *T. rangeli* and were unrelated to the diagnostic aspects and use of this species as an immunogen for *T. cruzi* infection.

We examined the ‘Abstract’ of each article and selected only those texts that highlighted the diagnostic implications in humans and/or comprised research on possible vaccines, developed using *T. rangeli*, for *T. cruzi* infection for a systematic review. After initial screening, all potentially relevant studies were downloaded in full text and evaluated for eligibility.

## RESULTS

Of the 122 articles retrieved by searching the descriptor "*Trypanosoma rangeli*", applying the filters that limited the subject to “Health Sciences” and “Human Studies” (84 by PubMed and 38 by SciELO), and eliminating duplicates (1 duplication), 121 publications were submitted for the evaluation of ‘Titles’, leaving only 50 papers for further evaluation. The ‘Abstracts’ of these papers were read, and 23 articles were selected for the composition of this review. However, 5 manuscripts could not be included in the study because they could not be downloaded in full text. Thus, 18 articles were selected for the present analysis.

Table 1 lists these texts in an ascending chronological order and gives information on the general profile of the articles that were being constructed. In this compilation of publications, (i) a time interval of 35 years between the most recent and oldest publications, (ii) research on both protozoa (*T. rangeli* and *T. cruzi*) in almost all texts (17 out of 18), (iii) a predominance of the type of descriptive experimental study in almost all texts (15 out of 18); research with human animal model (12 out of 18) and (iv) research in Latin American countries (Panama, Venezuela, Argentina, Colombia, Brazil, Chile, and Guatemala), belonging to the epidemiological distribution of Chagas disease, could be observed.

**TABLE 1:** Description of the qualitative data extracted from the 18 studies included in this review: characteristics of the publications: author, year, and country; characteristics of the studies: species, focus, type of study, and animal model.

Source	Country	Species	Focus	Type of study	Animal model
Anthony et al.,	Panama	*T. rangeli*,	Clinical Aspects	Experimental,	Mice,
(1979)^23^.		*T. cruzi*	Diagnoses	Descriptive	Humans
Schottelius et al.,	Germany	*T. cruzi*,	Clinical Aspects	Experimental,	Mice,
(1984)^22^.		*T. rangeli*	Diagnoses	Descriptive	Humans
Avila et al.,	Venezuela	*T. rangeli*,	Clinical Aspects	Brief communication	Humans
(1987)^24^.		*T. cruzi*	Diagnoses		
Guhl et al.,	London	*T. rangeli*,	Clinical Aspects	Case reports	Humans
(1987)^25^.		*T. cruzi*	Diagnoses		
Avila et al.,	Venezuela	*T. rangeli*,	Clinical Aspects	Experimental,	Humans
(1990)^30^.		*T. cruzi*	Diagnoses	Descriptive	
Ross et al.,	USA	*T. rangeli*,	Clinical Aspects	Experimental,	Humans
(1993)^26^.		*T. cruzi*	Diagnoses	Descriptive	
O'Daly et al.,	Venezuela	*T. rangeli*,	Clinical Aspects	Experimental,	Humans
(1994)^27^.		*T. cruzi*	Diagnoses	Descriptive	
Saldaña et al.,	Panama	*T. rangeli*,	Diagnoses	Experimental,	*In vitro*
(1996)^29^.		*T. cruzi*		Descriptive	
Vásquez et al.,	Panama	*T. rangeli*,	Epidemiological	Experimental,	Humans
(1997)^28^.	Sweden	*T. cruzi*	Diagnoses	Descriptive	
Tanoura et al.,	Japan	*T. rangeli*	Epidemiological	Experimental,	Mice, *In vitro,*
(1999)^19^.	Guatemala		Diagnoses	Descriptive	Humans
			Host-parasite		
			relationship		
Paláu et al.,	Colombia Chile	*T. rangeli*,	Vaccines against	Experimental,	Mice
(2003)^15^.		*T. cruzi*	*T. cruzi* infection	Descriptive	
Basso et al.,	Argentina	*T. rangeli*,	Vaccines against	Experimental,	Mice
(2008)^16^.		*T. cruzi*	*T. cruzi* infection	Descriptive	
Sousa et al.,	Brazil	*T. rangeli*,	Epidemiological	Case reports	Humans
(2008)^20^.		*T. cruzi*	Prophylactic		
			Clinical Aspects		
			Diagnoses		
Botero et al.,	Colombia	*T. rangeli*,	Diagnoses	Experimental,	*In vitro*
(2010)^31^.		*T. cruzi*		Descriptive	
Parada et al.,	Spain	*T. rangeli*,	Epidemiological	Letter to the Editor	Humans
(2010)^21^.		*T. cruzi*	Prophylactic		
			Clinical Aspects		
			Diagnoses		
Marini et al.,	Argentina	*T. rangeli*,	Vaccines against	Experimental,	Mice
(2011)^17^.		*T. cruzi*	*T. cruzi* infection	Descriptive	
Basso et al.,	Argentina	*T. rangeli*,	Vaccines against	Experimental,	Mice
(2014)^18^.		*T. cruzi*	*T. cruzi* infection	Descriptive	
Ferreira et al.,	Brazil	*T. rangeli*,	Diagnoses	Experimental,	Humans
(2014)^32^.		*T. cruzi*		Descriptive	Mice

Articles obtained from the systematic review process were grouped according to the publication date (ascending order) and classified according to qualitative variables.

Four articles identified the experiments proposed for the development of vaccines against *T. cruzi* infection by inoculating mice with *T. rangeli* strains^15^ ^,^ ^16^ ^,^ ^17^ ^,^ ^18^. Paláu et al. (2003)^15^ and Basso et al. (2008)^16^ demonstrated the association between the parasitemia levels and survival rates of the virulent *T. cruzi* populations in the control group and the group immunized with *T. Rangeli*. Even though Paláu et al. (2003)^15^ and Basso et al. (2008)^16^ utilized the metacyclic trypomastigote and epimastigote *T. rangeli* strains, respectively, both articles presented correlated results, underlining parasitemia reduction and increased survival rates of the immunized mice, compared to the control mice. Moreover, analysis of the histological preparations of the skeletal and cardiac muscles of the mice in the study by Basso et al. (2008)^16^ revealed that only moderate lymphomonocyte infiltrates were observed in the *T. cruzi*-infected vaccinated group, whereas many amastigote nests and severe inflammatory infiltrates were detected in the non-vaccinated group.

Marini et al. (2011)^17^ and Basso et al. (2013)^18^ conducted studies with a greater focus on understanding the immunobiochemical response patterns of *T. cruzi* infection in mice inoculated with *T. rangeli* strains. Marini et al. (2011)^17^ showed that the vaccination process promoted a highly adaptive response with specific IgG isotypes and the modulation of interleukin (IL)-6 levels during the early periods of infection, while Basso et al. (2013)^18^ showed that the vaccination process could be attributed to a strong innate immune response with important phagocytic activity. The latter ascertained the pertinence of macrophages in the recognition of *T. cruzi* epitopes, similar to those of *T. rangeli*, in the vaccine as an elementary aspect for the early elimination of *T. cruzi* and the low production of histological lesions in vaccinated mice.

It could be observed that 14 articles included in the systematic review showed a clinical, diagnostic, and/or epidemiological focus^19,20,21,22,23,24,25,26,27,28,29,30.31.32^. Tanoura et al*.* (1999)^19^investigated *T. rangeli* parasitemia, which was characterized by the gradual reduction of the acute infection, in human and murine hosts, suggesting that *T. rangeli* trypomastigotes could survive in the host’s blood for long periods without proliferating. Sousa et al. (2008)^20^ documented the isolation, by blood culture, and characterization of the *T. rangeli* stocks from two chronic chagasic patients and confirmed the identification of *T. rangeli* by visualizing the parasite in laboratory samples. Parada et al*.* (2010)^21^ documented the first case of *T. rangeli* infection in a blood donor in Europe, concluding that the possibility of *T. rangeli* infection should also be considered in patients with a history of previous positive tests, even if antibody serology for Chagas disease was negative. Additionally, 11 of these articles showed greater emphasis on improving the techniques and strategies of diagnostic differentiation of *T. rangeli* and *T. cruzi* infections^22^ ^,^ ^23^ ^,^ ^24^ ^,^ ^25^ ^,^ ^26^ ^,^ ^27^ ^,^ ^28^ ^,^ ^29^ ^,^ ^30^ ^,^ ^31^ ^,^ ^32^. 

Schottelius et al*.* (1984)^22^ showed that according to the different lytic effects manifested in mouse and human serum, lectin tests, in combination with complementary lysis, could be useful in distinguishing such protozoa.

On the other hand, considering the possibility of cross-reactivity of *T. rangeli* with *T. cruzi* in laboratory tests, Anthony et al*.* (1979)^23^ evaluated the use of the micro-ELISA technique in 229 residents of a Panamanian village, where both species were endemic. Avila et al*.* (1987)^24^ analyzed the antibodies in *T. rangeli-*infected patients and showed the presence of similar antibodies in Chagas disease patients and serum of *T. rangeli*-infected patients taken for serological diagnoses.

Furthermore, Guhl et al*.* (1987)^25^ reported the laboratory studies of 20 patients from the Rio Negro Valley, Colombia, demonstrating that 14 of them showed antibody reactions that were compatible with *T. cruzi* or *T. rangeli* infections in immunoassays. Of these patients, 4 were diagnosed with *T. rangeli* infection, 4 showed mixed infections, and 6 were infected with only *T. cruzi*. Ross et al*.* (1993)^26^ reported results of ELISA, immunofluorescence, and indirect hemagglutination tests conducted for 48 adults and warned that ELISA might not be the best choice of tests for epidemiological research on *T. cruzi*.

O'Daly et al*.* (1994)^27^studied the antibody response to these two protozoa and obtained the laboratory data on ELISA and immunoblotting with antigens of both species of chagasic patients. They observed that the humoral immune response to trypanosome antigens was complex and some chagasic serum reacted more strongly against *T. rangeli* than against *T. cruzi.* Moreover, Vásquez et al*.* (1997)^28^ reported the findings of immunohemagglutination tests, indirect immunofluorescence assay, and ELISA conducted for 65 individuals from an area endemic to *T. cruzi* and *T. rangeli* in a survey, demonstrating a greater serological immunoreactivity to *T. rangeli* preparations than to *T. cruzi* preparations in the studied population.

To establish a more specific form of laboratory diagnosis, other tests were also studied. Saldaña & Souza et al*.* (1996)^29^ discussed the possibility of the 43 kDa antigen being a specific marker for *T. rangeli*. Avila et al*.* (1990)^30^ identified high levels of natural anti-cerebroside (antiC) IgM antibody in *T. rangeli*-infected patients (56% of the patients); however, antiC IgM antibodies were also present in patients with chronic chagasic disease (30%) and human visceral (57%) and cutaneous (20%) leishmaniasis.

Botero et al*.* (2010)^31^ demonstrated a method used for diagnostic differentiation of both protozoa, noting that polymerase chain reaction (PCR) and minicircle amplification, followed by DNA probe hybridization and restriction fragment length polymorphism (RFLP) analysis, could improve the diagnosis of Chagas disease. Working with similar tools, Ferreira et al. (2014)^32^ investigated specific markers of *T. rangeli* species to identify intraspecific polymorphisms and target PCR diagnostic methods, outlining the several primers capable of categorically recognizing the genomic DNA of both species. This seemed to be an efficient method for the identification of these protozoa during the acute and chronic phases of infection in vertebrate hosts and vectors, respectively^33^.

## DISCUSSION

The proposed area of study in this systematic review was focused on the significance of *T. rangeli* analysis in the medical field, encompassing queries about zoonosis, and the formulation of a suitable vaccine for Chagas disease. Both *Trypanosoma* species not only share vectors and habitat, but also show strong similar antigenic responses^6^ ^,^ ^34^. Thus, we sought to collect and evaluate data on the relationship between the protist and human beings from the main publications on *T. rangeli*.

Animal testing revealed that *T. rangeli* infection might elicit a humoral and/or cellular immune response and partial protection against subsequent *T. cruzi* infection^18^. A study that assessed *T. rangeli* inoculation of domestic dogs in rural areas of Argentina demonstrated the induction of an important antibody response against *T. cruzi* over prolonged periods and a significant reduction in parasitemia in the inoculated dogs. This suggested that antibodies against *T. rangeli* might be involved from the inception of *T. cruzi* elimination^6^. Thus, another favorable future study could include the determination and evaluation of protective antigens in isolated trials.

Though *T. rangeli* does not induce acute parasitemia in humans on its own, the understanding of its morphological forms of infection is advantageous to differentiate it from *T. cruzi* in vertebrate and invertebrate species^35^ ^,^ ^36^.

The disease is transmitted to vertebrate hosts mainly through the bite of invertebrate reservoirs, such as *R. prolixus*. The metacyclic trypomastigotes are inoculated when the arthropod feeds on the host’s blood^3^ ^,^ ^37^ ^,^ ^38^. *T. rangeli* demonstrated pathogenicity in experiments with this vector^39^ ^,^ ^40^ ^,^ ^41^. Tanoura et al. (1999)^42^ investigated the parasitic infection process of *T. rangeli* in the hosts and evaluated the metacyclogenesis process *in vitro* and the terminus of metacyclic trypomastigotes after infection in mice. They also examined fibroblast cultures, establishing that the parasitemia process was characterized by a gradual reduction of the acute infection and the hemolytic trypomastigotes could survive in the blood for long periods without proliferating.

Regarding the etiopathological diagnosis of both protozoa in Chagas disease patients, strategies for differentiating these parasites are required. The data obtained indicated that although ELISA was sufficiently sensitive for sero-epidemiological studies, the serological cross-reactivity between the two species was a possibility that must be considered, even in the cases of double infection. Ross et al. (1993)^26^ emphasized that, on epidemiological scrutiny, the ELISA test would not be the best choice of tests for *T. cruzi* infection due to possible cross-reactions.

Together, the referenced articles were the result of meticulous analyses of the pertinent studies related to *T. rangeli* infections in humans. *T. rangeli* strains inoculated in mice appeared to be favorable stimuli for innate immune response (critical phagocytic macrophage activity, production of specific IgG isotypes, and modulation of IL-6 levels) against the subsequent *T. cruzi* inoculations. We underlined a few notable aspects including, the significant reduction in parasitemia, high survival rates of the immunized mice compared to the control organisms, and reduction in the infectious and inflammatory processes of *T. cruzi* in the groups immunized with *T. rangeli* ^31^ ^,^ ^32^. Moreover, these species showed several similarities in their geographical distributions, morphological aspects, and antigenic responses, and this, in hindsight, could be advantageous (preparation of efficient vaccine formulations) and/or disadvantageous (adequate diagnosis and treatment).

It was also noted that PCR and minicircle amplification, followed by DNA probe hybridization and RFLP analysis, were viable alternatives for more specific diagnosis^31^. However, since they are experimental methods, they may not be very accessible and/or feasible in regions with higher incidence on infection, given the lack of financial resources directed to public health sectors. 

## CONCLUSIONS

Our research could be a preliminary study and catalyst for more in-depth analyses in this area. Additionally, we emphasize the need for future experimental investigations to clarify the possibility of using *T. rangeli* strains for the diagnosis and immunoprophylaxis of *T. cruzi* infection.
